# Low-dose irradiation of mouse embryos increases Smad-p21 pathway activity and preserves pluripotency

**DOI:** 10.1007/s10815-018-1156-y

**Published:** 2018-03-16

**Authors:** Masami Hayashi, Kayo Yoshida, Kohei Kitada, Akane Kizu, Daisuke Tachibana, Mitsuru Fukui, Takashi Morita, Masayasu Koyama

**Affiliations:** 1 Department of Obstetrics and Gynecology, Osaka City University, Graduate School of Medicine, 1-4-3 Asahimachi, Abeno-ku, Osaka, 545-8585 Japan; 2 Department of Molecular Genetics, Osaka City University, Graduate School of Medicine, 1-4-3 Asahimachi, Abeno-ku, Osaka, 545-8585 Japan; 3 Laboratory of Statistics, Osaka City University, Graduate School of Medicine, 1-4-3 Asahimachi, Abeno-ku, Osaka, 545-8585 Japan

**Keywords:** Preimplantation embryo development, X-ray irradiation, Apoptosis, Pluripotency, Embryo culture

## Abstract

**Purpose:**

To study the outcomes of mouse preimplantation embryos irradiated with low doses of X-rays (≤ 1 Gy) and investigate apoptosis and pluripotency of the irradiated embryos.

**Methods:**

Mouse embryos at the 2-cell stage were collected for in vitro culture. After reaching the 8-cell stage, embryos were irradiated with various low doses of X-rays (0–1 Gy). Blastocysts with a normal appearance were transferred into a pseudopregnant uterus. The developmental rate to blastocysts and the survival rate following embryo transfer were examined. Expression levels of p21, Smad2, Foxo1, Cdx2, Oct4, and Nanog genes were measured by RT-PCR. Apoptotic cells in mouse blastocysts were examined immunofluorescently by staining for cleaved caspase-3.

**Results:**

More than 90% of non-irradiated and low-dose X-ray-irradiated preimplantation embryos developed to morphologically normal blastocysts that could be implanted and survive in the uterus. However, embryos irradiated with X-rays had more apoptotic cells in a dose-dependent manner. Expression of p21, Smad2, and Foxo1 genes in X-ray-irradiated embryos was increased significantly, while expression of Cdx2, Oct4, and Nanog genes was maintained in comparison with non-irradiated embryos.

**Conclusions:**

Although irradiated embryos contained apoptotic cells, the low doses of irradiation did not disturb development of 8-cell stage embryos to blastocysts or their survival in utero. The underlying mechanisms might involve anti-apoptotic systems, including the Smad-p21 pathway, and preservation of pluripotency.

**Electronic supplementary material:**

The online version of this article (10.1007/s10815-018-1156-y) contains supplementary material, which is available to authorized users.

## Introduction

Radiation is indispensable for the diagnosis of diseases. Imaging techniques, such as X-rays, computed tomographic scans, and position emission tomography, are widely used in clinical practice [1, 2]. Radiation is also applied for therapeutic methods including anti-oncogenic treatment of various organs [3]. However, clinicians should keep in mind that women of fertile age have the potential to carrying children and should consider the so called ten days rule after menstruation when using radiation techniques for diagnosis and/or treatment.

Irradiated preimplantation embryos either die or survive without any detectable malformation [4], and the effect of irradiation on an embryo depends on both the embryo stage and exposure dose [5]. When the fetal radiation dose is less than 0.05 Gy, non-carcinogenic risk such as abortion and malformation is negligible. The effect of a fetal dose of less than 0.1 Gy will be clinically not detectable [6] It has been suggested that minimal threshold of the central nervous system may be 0.06–0.3 Gy [7]. Excess of 1-Gy exposure during embryogenesis will result in clinically significant fetal damage. Exposition dose by typical imaging of a single phase of pelvic CT scan would reach 0.01–0.05 Gy and the level will not be considered as a reason to terminate pregnancy [6]. However, several times of examination of mixed X-irradiation will possibly come over 0.1 Gy and radiation effect may be not negligible. So, we studied the embryonic effects of 0 and 0.1–1.0 Gy by X-irradiation in the molecular level.

In response to double-strand breaks of DNA induced by radiation, ATM (ataxia telangiectasia mutated) is recruited to phosphorylate the tumor suppressor protein p53 [7, 8]. Phosphorylated p53 coordinates cell cycle arrest and apoptosis. In cell cycle arrest, p53 upregulates p21, a cyclin-dependent kinase (CDK) inhibitor [9]. p21 suppresses cyclin/CDK activity and binds to proliferating cell nuclear antigen, resulting in cell cycle arrest at G1 and G2 [10–12]. When the level of DNA damage is severe, unrepaired double-strand breaks (DSBs) can lead to apoptosis by p53-transcriptional regulation of genes with apoptotic activity [13].

Embryonic stem cells (ESCs), which are derived from the inner cell mass (ICM) of blastocysts, can undergo unlimited self-renewal and retain pluripotency to differentiate into all cell types of the body [14]. In contrast to somatic cells, which activate G1/S, intra S, and G2/M checkpoints after DSBs, ESCs lack the G1/S checkpoint and undergo only a temporary delay at G2/M phase in response to DSBs [15]. This property is thought to be a reason why ESCs are highly sensitive to radiation. Recent studies suggest that p21 might be involved in the apoptotic process of ESCs after radiation exposure, and p21 is activated through Smad and Forkhead box, class O (Foxo) pathways [16–19].

Despite the accumulated data from irradiated ESCs, we still lack information regarding effects of low-dose irradiation on embryos in terms of clinical medicine. Using 8-cell stage mouse embryos irradiated with a low dose (0.1–1.0 Gy), we aimed to (1) observe the developmental rate and survival rate after embryo transfer, (2) investigate the apoptotic process including the Smad-p21 pathway, and (3) evaluate the pluripotency of the ICM and trophectoderm (TE).

## Materials and methods

### Animals and embryos

Mice were maintained according to the Osaka City University guidelines for animal experimentation. BDF1 mice aged 8–10 weeks were superovulated by an intraperitoneal injection of 6.7 IU pregnant mare serum gonadotropin (PMSG; serotoropin, Aska Pharmaceutical Co.). Forty-eight hours after PMSG injection, the mice were injected with 6.7 IU human chorionic gonadotrophin (hCG; gonadotropin, Aska Pharmaceutical Co.). They were mated with BDF1 males of > 12 weeks of age. At 42–45 h after hCG injection, 2-cell embryos were collected from the oviducts by puncturing the ampulla portion of the oviduct with a needle in M2-buffered medium under a stereomicroscope. All embryos were cultured in a 100 μl drop of M16 medium covered by mineral oil at 37 °C in a humidified atmosphere with 5% CO_2_.

### X-ray irradiation

Twenty-four hours after collection of 2-cell stage embryos, development of the embryos was observed under the stereomicroscope. Embryos that had developed to the 8-cell stage were collected in 100 μl M2 medium. These 8-cell stage embryos were irradiated with X-rays at doses of 0.1, 0.5, and 1.0 Gy with dose rate of 0.7 Gy/min by an X-ray irradiation device (MBR-1520A-2; Hitachi Medical Corporation). After X-ray irradiation, the embryos were washed with M16 medium and cultured at 37 °C.

### Real-time reverse transcription polymerase chain reaction analysis

Thirty-three hours after X-ray irradiation, blastocysts with a normal appearance were collected in a pool and their RNA was extracted. More than 80 embryos were prepared for each dose condition (0, 0.1, 0.5, 1 Gy; total > 320 embryos) and irradiated. The morphologically normal 60 embryos of each dose were divided into three groups with 20 embryos. RNAs were extracted from 20 embryos. These blastocysts were washed in drops of phosphate-buffered saline (PBS) twice. RNA samples were isolated using an RNeasy Micro Kit (QIAGEN) and dissolved in 14 μl RNase-free water. The three RNA samples from each radiation condition were used for RT-PCR as triplicate. cDNA was synthesized from 10 μl RNA in a 10-μl reaction mixture with random primers and reverse transcription reagent using a High-Capacity cDNA Reverse Transcription Kit (Applied Biosystems). mRNA expression was analyzed by TaqMan real-time PCR. Reactions were carried out using TaqMan Gene Expression Master Mix (Applied Biosystems) in a final volume of 20 μl and performed in duplicate for each cDNA sample. Primer sets for 18S rRNA (Mm03928990_g1), p21 (Mm01212280_m1), Smad2 (Mm00487530_m1), Foxo1 (Mm00490671_m1), octamer-binding transcription factor 4 (Oct4; Mm03053917_g1), caudal-type homeobox protein 2 (Cdx2; Mm01212280_m1), and Nanog (Mm02019550_s1) were purchased from Applied Biosystems. Amplifications were performed under standard conditions by ABI7500 Fast System.

### Immunofluorescence staining

Embryos were washed, fixed with 4% paraformaldehyde in PBS for 20 min, permeabilized for 20 min at room temperature in PBS containing 0.5% Triton X-100, and then blocked in a 3 mg/ml bovine serum albumin solution for 30 min. They were immunostained with anti-γH2AX monoclonal antibody (1:1000 dilution, Novus biologicals), or anti-cleaved caspase-3 monoclonal antibody (1:400 dilution, Cell Signaling) overnight at 4 °C and then treated with a secondary antibody conjugated with an Alexa Fluor (goat anti-rabbit IgG secondary antibody, Alexa Fluor 488 conjugate, 1:1000 dilution; Thermo Scientific) and 6-diamidio-2-phyenylindole (DAPI) for 2 h at room temperature. The embryos were washed and observed under a fluorescence microscope. At least 10 embryos were analyzed for each (0, 0.1, 0.5, 1 Gy) irradiation.

### Implantation into a pseudopregnant uterus

ICR pseudopregnant mice (8–10 weeks old) at 2 days post-coitum (dpc) were purchased from Japan SLC. Ten blastocysts with a normal appearance at 33 h after X-ray irradiation were implanted in a pseudopregnant uterus (one side of a bi-corner uterus) using a glass capillary. The pregnant mice were examined at 19 dpc. The number of placentas, fetuses, and implantation marks was counted.

### Statistical analysis

Statistical analysis was performed using the exact *t* test, Student’s *t* test, one-way ANOVA, Williams test, Kruskal-Wallis test, and Shirley-Williams test. *p* < 0.05 was considered as significant.

## Results

### Most low-dose X-ray-irradiated preimplantation embryos develop to morphologically normal blastocysts in vitro

**Fig. 1:**
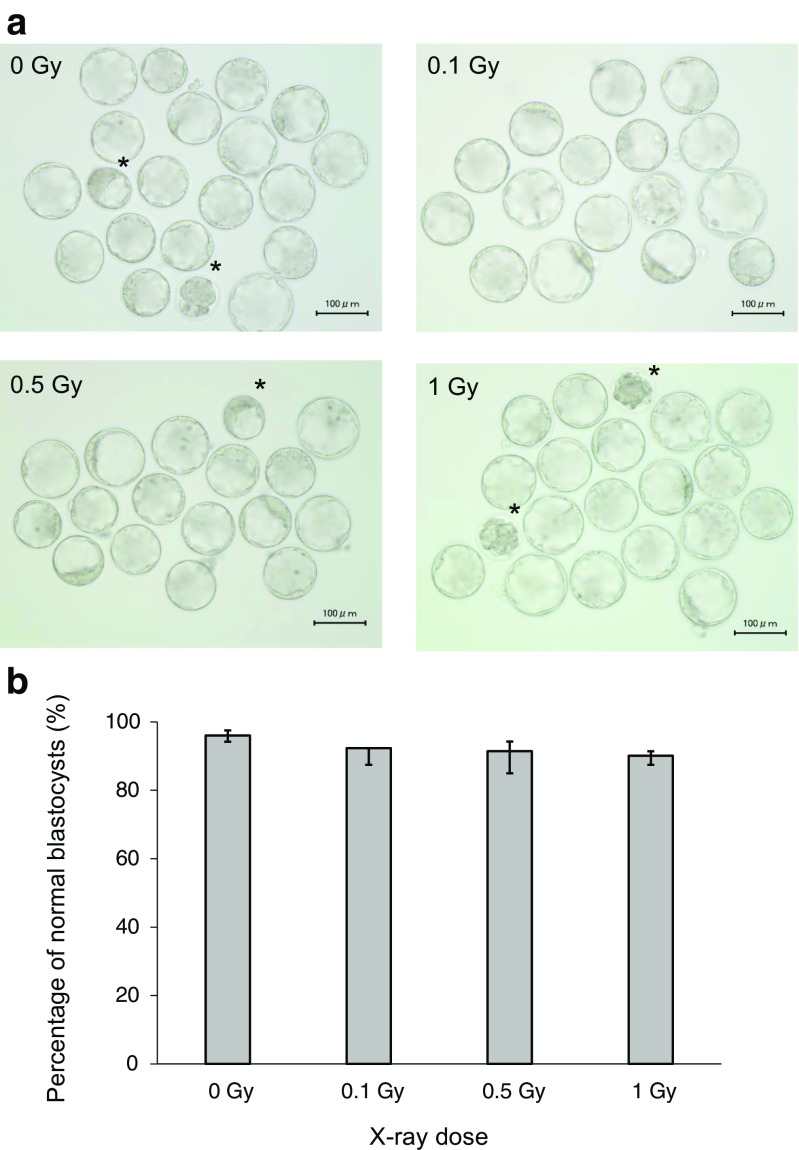
Development of preimplantation embryos irradiated with X-rays. **a** Representative micrographs of blastocysts with or without X-ray irradiation (0.1, 0.5, and 1.0 Gy). Asterisks indicate abnormal blastocysts. **b** The developmental rate of embryos irradiated with X-rays. Bars represent the maximum and minimum percentage of each experiment

A total of 96.1% of embryos without irradiation (0 Gy) developed to normal blastocysts (Table 1). The developmental rates of embryos irradiated with 0.1, 0.5, and 1.0 Gy to normal blastocysts were 92.3, 91.4, and 90.1%, respectively (Table 1 and Fig. 1b). Abnormal embryos that included developmentally delayed embryos by microscopic observation were indicated (Fig. 1a, asterisks). No significant difference was observed in the developmental rates to normal blastocysts by low doses of X-irradiation.

**Table 1:** Preimplantation embryo development after X-ray irradiation

Irradiation dose (Gy)	Number of embryos	Number of blastocyst^a^ (%)		Number of abnormal embryos^a^ (%)	
0	152	146	96.1%	6	3.9%
	(52, 40, 35, 25)^b^	(49, 39, 33, 25)^b^		(3, 1, 2, 0)^b^	
0.1	143	132	92.3%	11	7.7%
	(52, 40, 26, 25)^b^	(48, 35, 24, 25)^b^		(4, 5, 2, 0)^b^	
0.5	152	139	91.4%	13	8.6%
	(55, 40, 35, 22)^b^	(51, 34, 33, 21)^b^		(4, 6, 2, 1)^b^	
1.0	152	137	90.1%	16	9.9%
	(52, 40, 35, 25)^b^	(47, 35, 32, 23)^b^		(5, 2, 3, 2)^b^	

^a^The difference in the developmental rate was not significant among control, 0.1-, 0.5-, and 1.0-Gy-irradiated embryos by the exact *t* test

^b^Each number of embryos of four times experiments

### Blastocysts with a normal appearance after ≤ 1-Gy X-ray irradiation produce fetuses

To examine whether X-ray-irradiated embryos could develop in the uterus, we transferred 10 embryos that developed normally after X-ray irradiation into a pseudopregnant uterus (one side of the bi-corner uterus). We analyzed five pseudopregnant mice for each group: control and 0.1, 0.5, or 1.0 Gy-irradiated blastocysts. Non-irradiated embryos had a mean implantation number of 8.4 (implantation rate of 86%) as judged by the number of placentas and implantation marks with 7.7 becoming fetuses (Table 2). Embryos irradiated with 0.1 Gy had an implantation number of 8.7 with 6.5 becoming fetuses, those irradiated with 0.5 Gy had an implantation number of 8.6 with 6.0 becoming fetuses, and those irradiated with 1.0 Gy had an implantation rate of 8.1 with 6.7 becoming fetuses (Table 2). The irradiated embryos had implantation and fetal development rates similar to those of non-irradiated embryos.

**Table 2:** Pregnancy outcome of irradiation

Irradiation dose (Gy)	Number of pregnant mice analyzed	Number of implantations^a^ (average)	Number of fetuses^a^ (average)
0	5	8.4 ± 1.6	7.7 ± 1.6
0.1	5	8.7 ± 1.3	6.5 ± 2.0
0.5	5	8.6 ± 0.9	6.0 ± 1.1
1.0	5	8.1 ± 1.3	6.7 ± 1.9

^a^There was no significant difference in the number of implantations or fetuses between each group by the exact *t* test

### One-Gy irradiation induces DNA damage in embryos

**Fig. 2:**
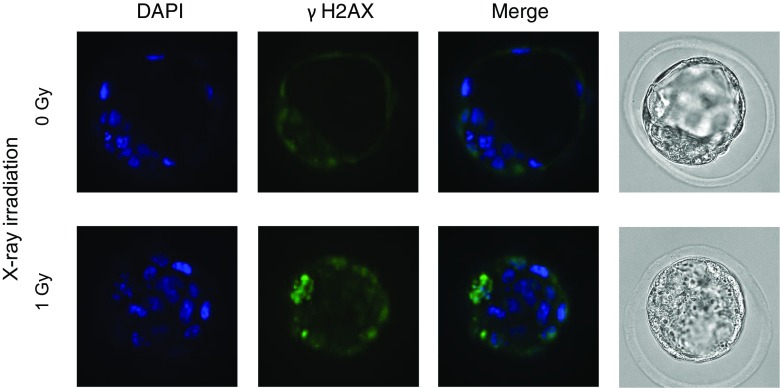
γH2AX expression in blastocysts after X-ray irradiation. Representative images showing the immunofluorescence intensity of γH2AX (green) in control (0 Gy) and 1-Gy X-ray-irradiated embryos. Merged micrographs show the localization of γH2AX (green) with DAPI staining (blue)

To determine the effect of low-dose irradiation (1 Gy), we evaluated DNA damage in morphologically normal blastocysts. Immunofluorescence staining by γH2AX revealed that at the blastocyst stage, the embryos without X-irradiation showed low staining by γH2AX, but at 1 Gy of irradiated blastocysts, several cells were strongly stained by γH2AX antibody. The embryos of 1 h after X-irradiation by 1 Gy were weakly stained by γH2AX antibody with non-specific signals in the cell surface (Supplemental Fig. 1), while some cells in blastocyst strongly stained after 33 h of X-irradiation as in Fig. 2. This suggested that the several significantly damaged cells in blastocyst might lead to apoptosis followed by DNA double-strand breaks, resulting in their strong phosphorylation of H2AX.

### Low-dose irradiation induces apoptosis in morphologically normal blastocysts

**Fig. 3:**
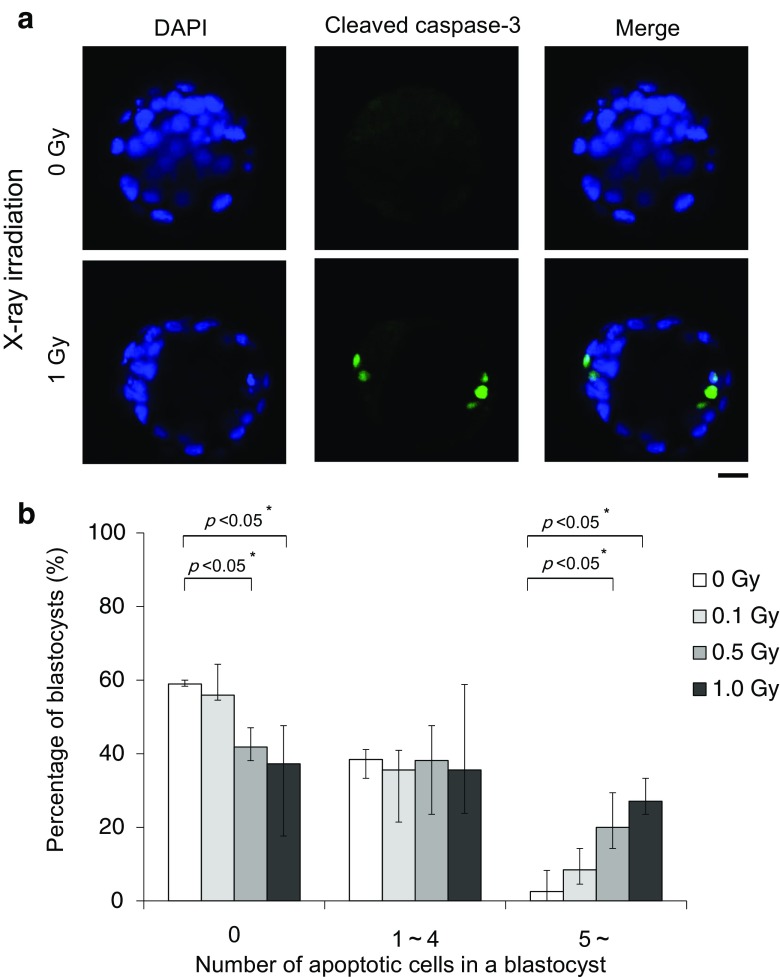
Cleaved caspase-3 expression in blastocysts after X-ray irradiation. **a** Representative images showing the immunofluorescence intensity of cleaved caspase-3 (green) in control (0 Gy) and 1-Gy X-ray-irradiated embryos. Merged micrographs show the localization of cleaved caspase-3 (green) with DAPI staining (blue). Bar = 20 μm. **b** Average percentages of apoptotic cell-containing blastocysts X-irradiated at 0, 0.1, 0.5, and 1 Gy are shown. Bars represent maximum and minimum percentages of each experiment. The immunofluorescence staining was repeated three times using more than 10 blastocysts in each dose

To determine the effect of low-dose irradiation, we evaluated apoptosis in morphologically normal blastocysts. Immunofluorescence staining of cleaved caspase-3 revealed that 1-Gy-irradiated embryos displayed more cells with caspase-3 immunoreactivity than non-irradiated embryos in the ICM and TE (Fig. 3a). After 1-Gy irradiation, 27.1% of blastocysts contained more than five apoptotic cells, 35.6% contained one to four apoptotic cells, and 37.3% did not contain any apoptotic cells (Fig. 3b and Table 3). After 0.5-Gy irradiation, 20% of blastocysts contained more than five apoptotic cells, 38.2% contained one to four apoptotic cells, and 41.8% did not contain any apoptotic cells. After 0.1-Gy irradiation, 8.5% of blastocyst contained more than five apoptotic cells, 35.6% contained one to four apoptotic cells, and 55.9% did not contain any apoptotic cells. In non-irradiated blastocysts, 2.6% of blastocysts contained more than five apoptotic cells, 38.5% contained one to four apoptotic cells, and 359.0% did not contain any apoptotic cells. Statistically, we analyzed the distributions by Kruskal-Wallis test and then compared the rates of apoptotic cell-containing cells by Shirley-Williams test. We judged the blastocysts without apoptotic cells were significantly (*p* < 0.05) decreased, when the embryos were irradiated at 0.5 and 1.0 Gy comparing with 0 Gy. On the contrary, the blastocysts with more than five apoptotic cells were significantly (*p* < 0.05) increased, when the embryos were irradiated at 0.5 and 1.0 Gy comparing with 0 Gy. These results confirmed that 0.5- and 1-Gy irradiation induced apoptosis in morphologically normal blastocysts.

**Table 3:** Number and percentage of apoptotic cells in a blastocyst

Irradiation dose (Gy)	Number of apoptotic cells in a blastocyst (%)						
	0		1–4		> 5		Total
0	23	59.0%	15	38.5%	1	2.6%	39
	(6, 10, 7)^a^		(4, 7, 4)^a^		(0, 0, 1)^a^		(10, 17, 12)^a^
0.1	33	55.9%	21	35.6%	5	8.5%	59
	(9, 12, 12)^a^		(4, 9, 8)^a^		(2, 1, 2)^a^		(15, 22, 22)^a^
0.5	23	41.8%	21	38.2%	11	20%	55
	(8, 8, 7)^a^		(4, 10, 7)^a^		(5, 3, 3)^a^		(17, 21, 17)^a^
1.0	22	37.3%	21	35.6%	16	27.1%	59
	(9, 10, 3)^a^		(5, 6, 10)^a^		(7, 5, 4)^a^		(21, 21, 17)^a^

There are significant differences between each group by Kruskal-Wallis test. Blastocysts without apoptotic cells were significantly (*p* < 0.05) decreased, when the embryos were irradiated at 0.5 and 1.0 Gy comparing with 0 Gy by Shirley-Williams test. On the contrary, the blastocysts with more than five apoptotic cells were significantly (*p* < 0.05) increased, when the embryos were irradiated at 0.5 and 1.0 Gy comparing with 0 Gy by Shirley-Williams test

^a^Each number of embryos of triplicate experiments

### Gene expression of p21, Smad 2, and Foxo1

**Fig. 4:**
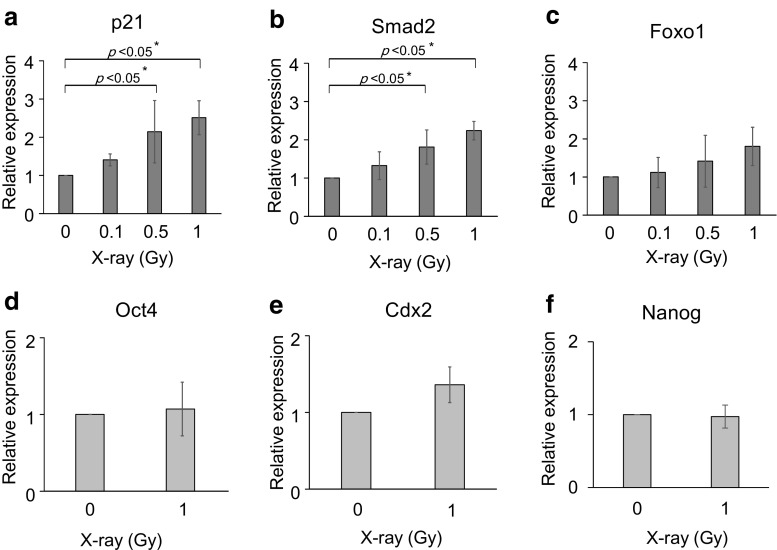
RT-PCR analysis of p21, Smad 2, Foxo1, Oct4, Cdx2, and Nanog mRNAs in blastocysts after low-dose irradiation. RT-PCR was conducted using three replicates of RNA samples from 20 X-ray-irradiated blastocysts (0.1, 0.5, and 1.0 Gy). The relative amount of mRNA was normalized to the 18S rRNA level and expressed the fold change compared the control sample. RT-PCR was conducted using three replicates of RNA samples. Error bars represent the standard error of the mean. *p < 0.05 versus control. **a** p21, **b** Smad 2, **c** Foxo1, **d** Oct4, **e** Cdx2, and **f** Nanog

Next, we examined gene expression of p21, which is related to cell arrest and apoptosis, and Smad 2 and Foxo1 that upregulate p21 by RT-PCR. The 8-cell stage embryos were irradiated and RNAs were extracted from normal blastocysts. We then analyzed p21, Smad 2, and Foxo1. Expression of p21 and Smad 2 genes was increased depending on the dose of irradiation. Normal blastocysts derived from 8-cell stage embryos irradiated with X-rays at 0.1 Gy had increased gene expression of p21 by 1.4-fold, compared with non-irradiated embryos (Fig. 4a). Normal blastocysts after X-ray irradiation at 0.5 and 1 Gy had significantly increased gene expression of p21 by 2.1-fold and 2.5-fold, respectively, compared with non-irradiated embryos (Fig. 4a). Normal blastocysts derived from 8-cell stage embryos irradiated with X-ray at 0.1 Gy had increased gene expression of Smad 2 by 1.3-fold, compared with non-irradiated embryos (Fig. 3b). The 0.5- and 1-Gy X-ray-irradiated normal blastocysts had significantly increased gene expression of Smad 2 by 2.2-fold and 2.8-fold, respectively, compared with non-irradiated embryos (Fig. 4b). Expression of the Foxo1 gene tended to increase in a dose-dependent manner, but it was not significant (Fig. 4c).

### Morphologically normal blastocysts after 1-Gy irradiation maintain developmental pluripotency

To determine the influence of irradiation on the pluripotency of embryos, we examined the gene expression of Cdx2, Oct4, and Nanog by RT-PCR. Normal blastocysts derived from 8-cell stage embryos irradiated with X-rays at 1 Gy did not show decreases in expression of Cdx2, Oct4, and Nanog genes (Fig. 4d–f). These results confirmed that the morphologically normal blastocysts retained pluripotency after irradiation at 1 Gy.

## Discussion

More than 90% of low-dose X-ray-irradiated preimplantation embryos developed into morphologically normal blastocysts that could be implanted and survive in the uterus similarly to non-radiated embryos, even though preimplantation embryos had more apoptotic cells compared with non-radiated embryos. This phenomenon might be due to preserved pluripotency as shown by maintained Oct4 and Nanog expression and increased activity of the Smad-p21 pathway that is thought to act as an anti-apoptotic factor.

Müeller et al. showed that 1-cell embryos had a dose-response relationship and increased frequency of lethal events (preimplantation deaths, resorptions, and fetal deaths) even at low-dose irradiation ranging from 0.25 to 1 Gy, whereas 32- to 64-cell embryos were unaffected by 1-Gy irradiation [20]. They also showed increased lethality in 32- to 64-cell embryos after 2-Gy irradiation, suggesting that a threshold may exist close to 1 Gy in multicellular embryos [20]. In frozen 8-cell mouse embryos, Glenister et al. found that even 2-Gy irradiation had no effect on their morphological appearance, development to blastocysts, implantation rate, or ratio of live fetuses to transferred embryos [21]. In this study, we collected and cultured 2-cell stage embryos in vitro and irradiated them with 1 Gy at the 8-cell stage. We observed that more than 90% of embryos developed to blastocysts, and the blastocysts successfully implanted and survived in the uterus similar to the control. Taken together with previous reports, our findings suggest that 1-Gy irradiation might have no effect on the development of multicellular embryos in culture and pregnant mice, or even frozen embryos.

p21 was originally thought to play an important role as a cell cycle regulator [10, 12]. More recently, it was found to play other fundamental roles including modulation of apoptosis [22–24]. Several studies have shown that suppression of p21 induces apoptosis in various human carcinoma cells [23, 24]. In preimplantation embryos, Niwa et al. studied sperm-irradiated embryos that were obtained from non-irradiated female mice mated with irradiated male mice exposed to 6-Gy X-rays [25]. They detected apoptotic cells in the ICM at the early blastocyst stage, but not at earlier stages. In addition, they found that apoptosis was more pronounced in sperm-irradiated p21^−/−^ embryos than that in wild-type p21 embryos. It was concluded that damage responses in preimplantation embryos occur in a stage-specific manner, and that p21 is involved in apoptosis at the blastocyst stage [25]. In this study, we first demonstrated an increase in p21 gene expression of low-dose-irradiated blastocysts. Considering the evidence reported by Suvorova et al. that p21 gene transcription increases gradually until day 5 [17], the prolonged effect of p21 might benefit blastocysts that have apoptotic cells to survive in the uterus.

The tumor suppressor gene p53 is a well-known transcriptional regulator of p21 [9]. In somatic cells, DNA damage by radiation upregulates p53 activity and subsequently results in p21 expression [9, 13, 26]. Norimura et al. reported that, after X-ray irradiation of pregnant mice at 3.5 days after conception (blastocyst stage) with 2 Gy, p53^+/+^ mice had no incidence of anomalies and a 73% incidence of death before completion of the placenta, whereas p53^−/−^ mice had a 22% incidence of anomalies and a 44% incidence of deaths before completion of the placenta. They suggested that embryos have a p53-dependent guardian mechanism that aborts cells bearing radiation-induced teratogenic DNA damage [27]. Furthermore, it has been reported that p21 is activated through a p53-independent pathway in somatic cells [28]. Transforming growth factor β (TGF-β) is a highly important growth factor in tissues after radiation exposure, which induces p21 through a p53-independent mechanism [29]. TGF-β induces upregulation of p21 via the Smad signaling pathway in response to DNA damage by radiation [18] and Foxo is known to act with TGF-β-activated Smad [19]. In the mouse embryo, Muñoz-Espin et al. showed that cellular senescence occurs during mouse embryonic development at the mesonephros and the endolymphatic sac of the inner ear. They observed that the senescence was dependent on p21, but independent of p53. In addition, they found that the expression of p21 was regulated by TGF-β/ SMAD and PIK3/FOXO pathways [30]. Our results suggested that low-dose irradiation upregulated the Smad-p21 pathway.

Blastocysts consist of the TE, the single outer cells involved in implantation, which expand to form extraembryonic tissues including the placenta, and the ICM, the inner cells that will develop into pluripotent progenitors of all fetal cell types [31]. Differentiation of the TE and ICM is regulated by several genes. Cdx2 is essential for expansion of the TE lineage [32], whereas Oct4, a member of the POU family of transcription factors and Nanog, a highly divergent homeodomain-containing protein, maintain pluripotency of the ICM [32–35]. Momcilovic et al. reported that, in human ESCs, a decrease in the mRNA level of Oct4 at 6 h following 2-Gy irradiation returned to the level observed in non-irradiated cells after 24 h, suggesting that the surviving human ESCs remained pluripotent after irradiation [36]. We showed that expression of Oct4, Cdx2, and Nanog was maintained in irradiated blastocysts at the same level as that in non-irradiated blastocysts. These results suggest that the surviving cells in low-dose-irradiated blastocysts maintain pluripotency of the ICM and the ability to expand the TE.

In conclusion, we showed that low-dose (0.1–1.0 Gy) irradiation did not disturb development of 8-cell stage embryos to blastocysts that survive in the uterus, even though the irradiated embryos included apoptotic cells in the ICM and TE. The underlying mechanism of our observation might be involved in the anti-apoptotic system including the Smad-p21 pathway as well as preserved pluripotency by Cdx2 and Oct4 expression to recover the damage, and develop and survive normally in subsequent embryological steps. These findings may be important for doctors and clinical embryologists in the field of reproductive medicine. Our findings are important to understand the mechanism of embryo development after low-dose irradiation and increase our understanding of the “all or none” phenomenon. Further studies are needed to elucidate the effect on malformation, and functional and neurological development of fetuses at risk of irradiation at the embryo stage.

## Electronic supplementary material

Supplemental Fig. 1 γH2AX expression in 8-cell stage embryo after X-ray irradiation. a Representative images showing the immunofluorescence intensity of γH2AX (green) in control (0 Gy) and 1 Gy X-ray-irradiated embryos. Merged micrographs show the localization of (green) with DAPI staining (blue). **b** Representative images showing the immunofluorescence intensity (green) in control (0 Gy) and 1 Gy X-ray-irradiated embryos without anti-γH2AX antibody. Merged micrographs are shown. (PPTX 21616 kb)
