# Supplementary figures and images for: Low-dose irradiation of mouse embryos increases Smad-p21 pathway activity and preserves pluripotency
Source: J Assist Reprod Genet. 2018 Mar 16;35(6):1061–9. doi: 10.1007/s10815-018-1156-y (PMC6030001; doi:10.1007/s10815-018-1156-y)

## Slide 1
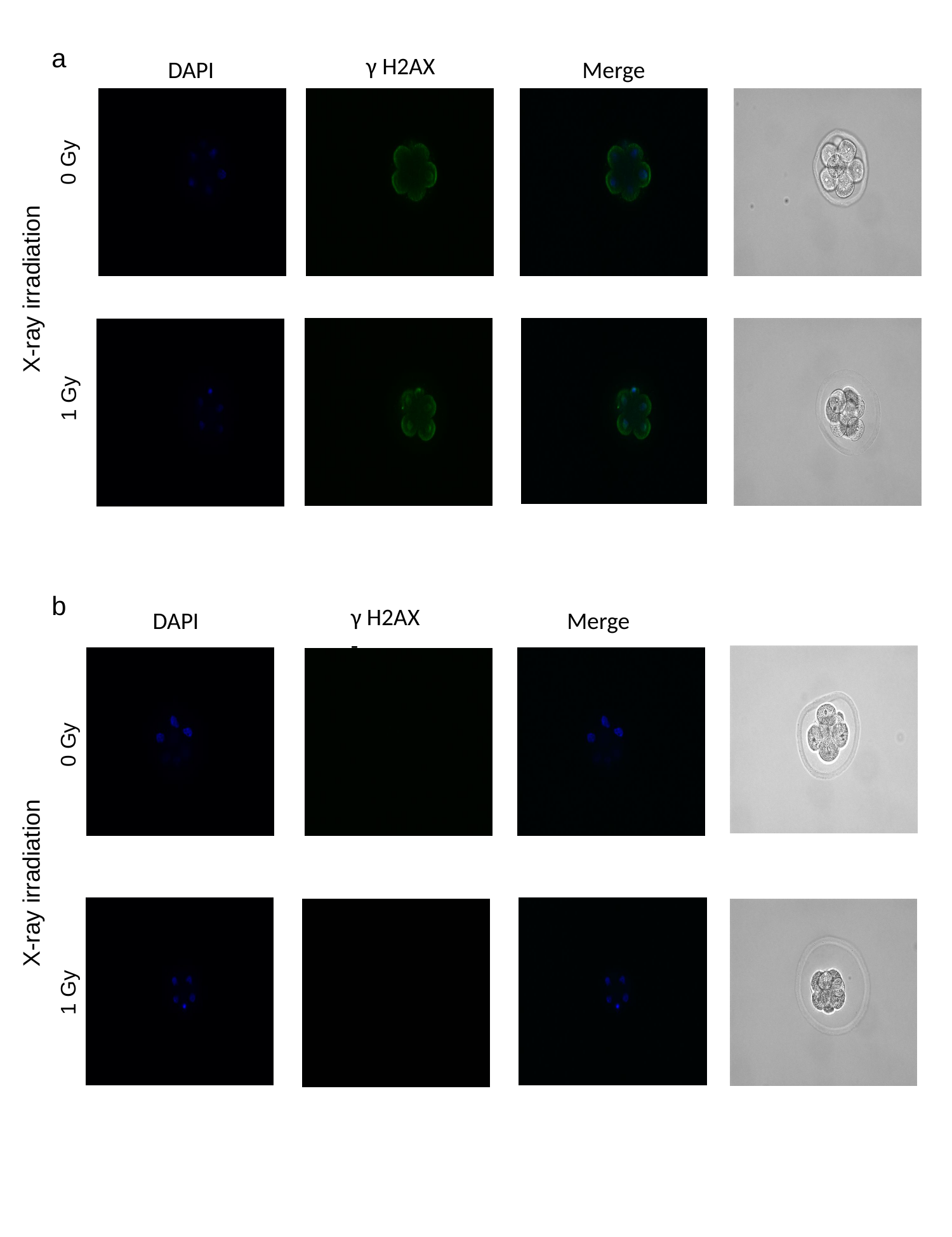

a
γ H2AX
DAPI
Merge
0 Gy
X-ray irradiation
1 Gy
b
γ H2AX　-
DAPI
Merge
0 Gy
X-ray irradiation
1 Gy

Supplement: Supplementary file 1 — γH2AX expression in 8-cell stage embryo after X-ray irradiation. a Representative images showing the immunofluorescence intensity of γH2AX (green) in control (0 Gy) and 1 Gy X-ray-irradiated embryos. Merged micrographs show the localization of (green) with DAPI staining (blue). b Representative images showing the immunofluorescence intensity (green) in control (0 Gy) and 1 Gy X-ray-irradiated embryos without anti-γH2AX antibody. Merged micrographs are shown. (PPTX 21616 kb) [file 10815_2018_1156_MOESM1_ESM.pptx]
